# Assessing the inflammation in pediatric MOGAD: Significance of CSF HMGB1 and related biomarkers

**DOI:** 10.3389/fimmu.2025.1534172

**Published:** 2025-02-06

**Authors:** Xin Wang, Ruibin Zhao, Jiayu Fan, Chong Liu, Li Zhang, Huafang Yang, Weiyi Wang

**Affiliations:** ^1^ Second Department of Neurology, Hebei Children’s Hospital, Shijiazhuang, China; ^2^ School of Medical Imaging, Hebei Medical University, Shijiazhuang, China; ^3^ Department of Pathology, Hebei Children’s Hospital, Shijiazhuang, China

**Keywords:** NLRP3, HMGB1, IL-6, MOGAD, pediatric, CSF

## Abstract

**Background and purpose:**

Myelin-oligodendrocyte glycoprotein antibody associated disease (MOGAD) is a common inflammatory disease of the central nervous system (CNS) in children that can lead to demyelination. Evaluation and monitoring of biomarkers associated with its pathogenesis would provide vital information on disease progression and therapeutic assessment.

**Methods:**

We assessed NLRP3, HMGB1, IL-6, and IL-33 levels in the cerebrospinal fluid (CSF) of pediatric patients with MOGAD at different time points and their association with the risk of disease. We recruited 30 patients with MOGAD (20 in the acute phase and 10 in remission) and 10 control patients with noninflammatory demyelinating disease. The expanded disability status scale (EDSS) was used to assess disease severity.

**Results:**

NLRP3, HMGB1, and IL-6 levels in the CSF were significantly higher in patients with MOGAD during the acute phase than in remission (*P* < 0.05, *P* < 0.05, *P* < 0.05) and the control group (*P* < 0.01, *P* < 0.0001, *P* < 0.01). HMGB1 levels were significantly correlated with NLRP3 levels (*P* < 0.01) during the acute phase. Moreover, we found notable correlation between HMGB1 levels and EDSS (*P* < 0.05) scores. IL-6 levels were significantly correlated with the total number of attacks (*P* < 0.05), but not with EDSS scores.

**Conclusions:**

These findings suggest that NLRP3, HMGB1, and IL-6 in the CSF may be potential therapeutic targets and are at least partly involved in the pathogenesis of pediatric MOGAD. HMGB1 in the CSF may be a potential biomarker correlating with pediatric MOGAD severity. Further investigations are warranted to validate potential cytokine pathways between that NLRP3, HMGB1, and IL-6 of MOGAD.

## Introduction

Myelin-oligodendrocyte glycoprotein antibody associated disease (MOGAD) is a recently discovered independent-spectrum disease associated with MOG-IgG antibodies (1). Some patients with MOGAD often present with highly recurrent courses, and repeated episodes may lead to long-term neurological dysfunction. Indicators associated with the pathogenesis and severity of MOGAD have not yet been fully understood.

The NOD-like receptor pyrin domain containing 3 (NLRP3) inflammasome plays a key role in mediating the expression and activation of inflammatory factors. Once activated, NLRP3 can recruit and cut pre-caspase-1, assist in the release of inflammatory factors, such as interleukin (IL), and ultimately trigger inflammatory response (2). Compared to healthy individuals, NLRP3 is overexpressed in mononuclear cells of patients with neuromyelitis optica spectrum disorders (NMOSD) and multiple sclerosis (MS) (3, 4). Additionally, certain risk factors, such as high mobility box 1 (HMGB1), can bind to inflammasome receptors to promote NLRP3 activation (5).

HMGB1 is a nucleoprotein involved in transcriptional regulation of nucleosome tissue, which plays a key role in inflammation. The released HMGB1 can accelerate the release of proinflammatory factors, such as IL-1β, IL-6, TNF-α, and trigger inflammatory response (6). IL-6, a proinflammatory factor, induces B and T cell differentiation and antibody production, and is significantly elevated of the central nervous system (CNS) (7). IL-33 is mainly expressed in oligodendrocytes (8). However, its role in the CNS remains unclear.

Herein, we evaluated NLRP3, HMGB1, IL-6, and IL-33 levels in cerebrospinal fluid (CSF) of pediatric patients with MOGAD both the acute phase and remission, for the first time, as well as their association with disease severity and compared them with controls.

## Patients and methods

### Participants

In this study, we examined 30 pediatric patients with MOGAD (20 in the acute phase and 10 in remission) and 10 age, weight, and sex-matched controls with nondemyelinating disease [febrile convulsion (*n* = 3), infectious meningitis (*n* = 2), migraine (*n* = 5)] at the Department of Neurology of Hebei Children’s Hospital. The clinical diagnosis of MOGAD was based on clinical manifestations, CSF and blood examinations, comprehensive neurological assessments, and brain magnetic resonance imaging (MRI) and the criteria proposed by the International MOGAD Panel (9). MOG-IgG titer was measured using fixed-CBA. Lumbar punctures were required for diagnosis or treatment for all the patients. Neurological impairment was evaluated using the extended disability status scale (EDSS). Clinical data and EDSS scores were obtained from two neurology clinicians of out-team. The study was approved by the Ethics committee of Hebei Children’s Hospital, and informed consent was obtained from all the patients prior to participation.

### Preparation of CSF samples

The CSF samples were collected during the acute phase, in remission, and from controls. The samples were stored at −80°C until further analysis. All procedures were completed in 30 min.

### Enzyme-linked immunosorbent assay

NLRP3 (ElAab, Wuhan, China) and HMGB1 levels (Elabscience, Wuhan, China) in CSF samples were quantified using enzyme-linked immunosorbent assay (ELISA) kits, according to the manufacturer’s instructions. IL-6 and IL-33 levels were also detected via ELISA (Multisciences, Zhejiang, China).

### Clinical evaluation

Clinical manifestations, blood tests, CSF analysis, and MRI scans were reviewed by professional neurologists to evaluate disease severity for each patient with MOGAD, and EDSS scores and total numbers of attacks were recorded.

### Statistical analyses

All results were analyzed using the Statistical Package for the Social Sciences (SPSS) 23.0. The datasets were first examined for normality and homogeneity of variance. Data are presented as mean ± standard deviation or percentage. The T-test was used to analyze differences between groups, whereas one-way analysis of variance was used to analyze differences among multiple groups. Correlations among profiles were assessed using Spearman’s rank analysis. *P* < 0.05 was considered statistically significant for all tests.

## Results

### Demographic and clinical data

The study recruited a total of 30 pediatric patients with MOGAD (female: *n* = 16 and male: *n* = 14), with a median age of 8.0 (6.0–10.0) years. The median number of attacks was 1 (1, 2), and the EDSS score was 1.5 (1.5–2.0). The average days of hospitalization was 21.5 (16.5–24.3). Twenty-three patients (23/30, 76.7%) received first-line immunotherapy, whereas the remaining seven (7/30, 23.3%) received escalation therapy. Age at sampling, gender, preceding events, white blood cells of CSF and CSF proteins were not significantly different for the different groups (**Table 1**).

**Table 1:** Relationship between basal characteristics and biomarkers in CSF of pediatric MOGAD.

Basal characteristics	MOGAD patients	Control patients	*P* value
Gender *n* (%)			
Male	14 (46.7)	5 (50)	0.643
Female	16 (53.3)	5 (50)	
Age at sampling (years)	7.67 ± 2.67	6.70 ± 3.40	0.360
Preceding event *n* (%)			
Respiratory infection or vaccination	20 (66.7)	6 (60)	0.492
none	10 (33.3)	4 (40)	
White blood cells of CSF	6.34 ± 4.06	4.30 ± 1.89	0.136
Protein of CSF	0.39 ± 0.09	0.33 ± 0.06	0.053

### NLRP3, HMGB1, and IL-6 levels in CSF increased in patients with MOGAD compared with patients in remission and controls

**Figure 1:**
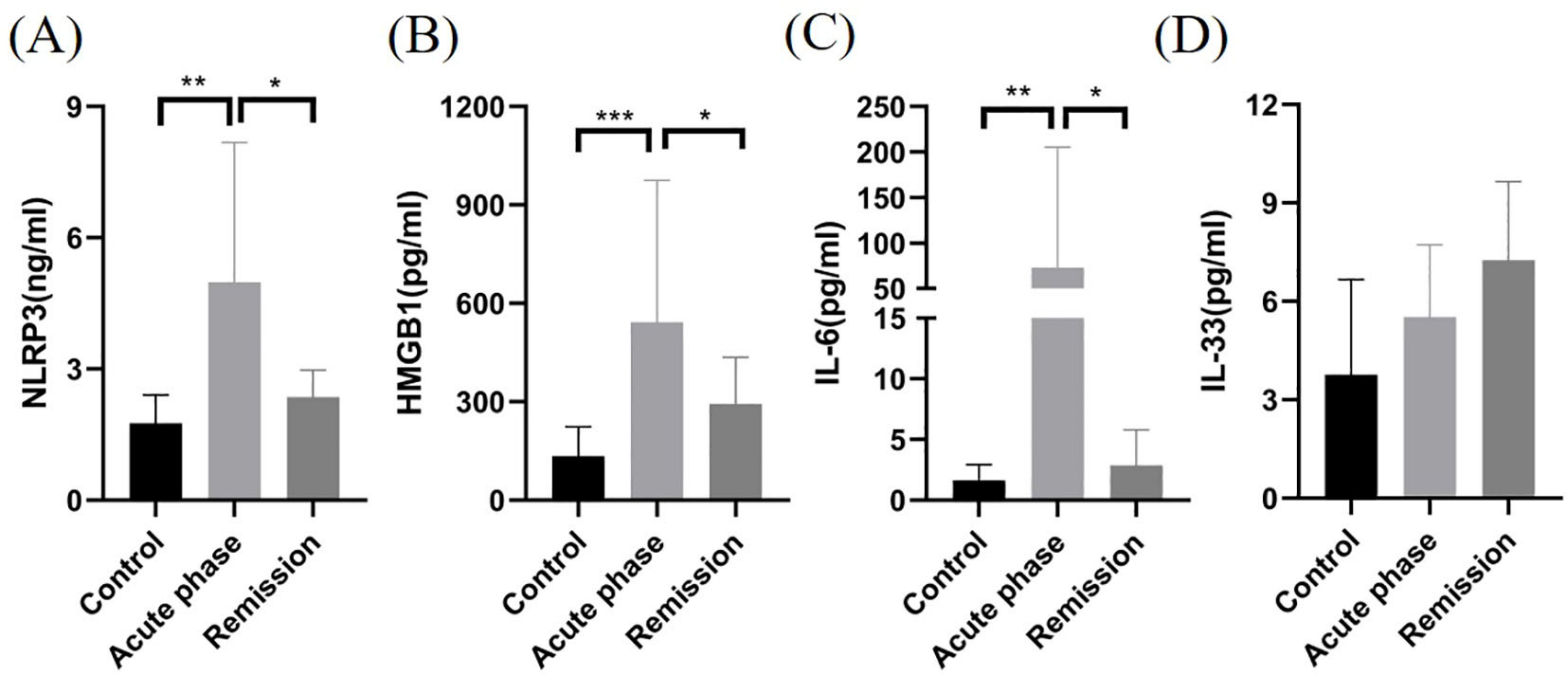
Comparison of NLRP3, HMGB1, IL-6, and IL-33 levels in CSF samples of patients with MOGAD during the acute stage, in remission, and with controls. NLRP3 **(A)**, HMGB1 **(B)**, and IL-6 **(C)** levels in the CSF of patients with MOGAD during the acute stage were significantly higher than those in the controls and remission (*P < 0.05, **P < 0.01, ***P < 0.001). No significant difference was observed in IL-33 **(D)** levels in the CSF of patients with MOGAD at different time points (*P*>0.05).

NLRP3 inflammasome (4.98 ± 3.20 and 1.76 ± 0.65, *P* < 0.01), HMGB1 (542.1 ± 433.0 and 134.3 ± 88.9, *P* < 0.001), and IL-6 (72.8 ± 13.3 and 1.63 ± 1.30, *P* < 0.01) levels significantly increased in CSF samples of patients with MOGAD during acute stages compared with noninflammatory demyelinating control patients (**Table 2**). Moreover, NLRP3 inflammasome (4.98 ± 3.20 and 2.36 ± 0.62, *P* < 0.05), HMGB1 (542.1 ± 433.0 and 293.1 ± 142.7, *P* < 0.05), and IL-6 (72.8 ± 13.3 and 2.87 ± 2.92, *P* < 0.05) levels markedly increased during acute stages compared with MOGAD remission stages (**Figures 1A–C**). However, no significant difference was observed in IL-33 levels in the CSF of patients with MOGAD at different timepoints (*P*>0.05) (**Figure 1D**).

**Table 2:** Comparison of biomarkers in different phases and groups.

	Acute stages of MOGAD	Remission stages of MOGAD	*P* value	Control patients	*P* value
NLRP3 (ng/ml)	4.98 ± 3.20	2.36 ± 0.62	0.035	1.76 ± 0.65	0.002
HMGB1 (pg/ml)	542.1 ± 433.0	293.1 ± 142.7	0.039	134.3 ± 88.9	0.0007
IL-6 (pg/ml)	72.8 ± 13.3	2.87 ± 2.92	0.017	1.63 ± 1.30	0.001
IL-33 (pg/ml)	5.52 ± 2.21	7.26 ± 2.40	0.067	3.76 ± 2.90	0.061

### Relationship between inflammatory factors in the CSF of patients with MOGAD during the acute phase

**Figure 2:**
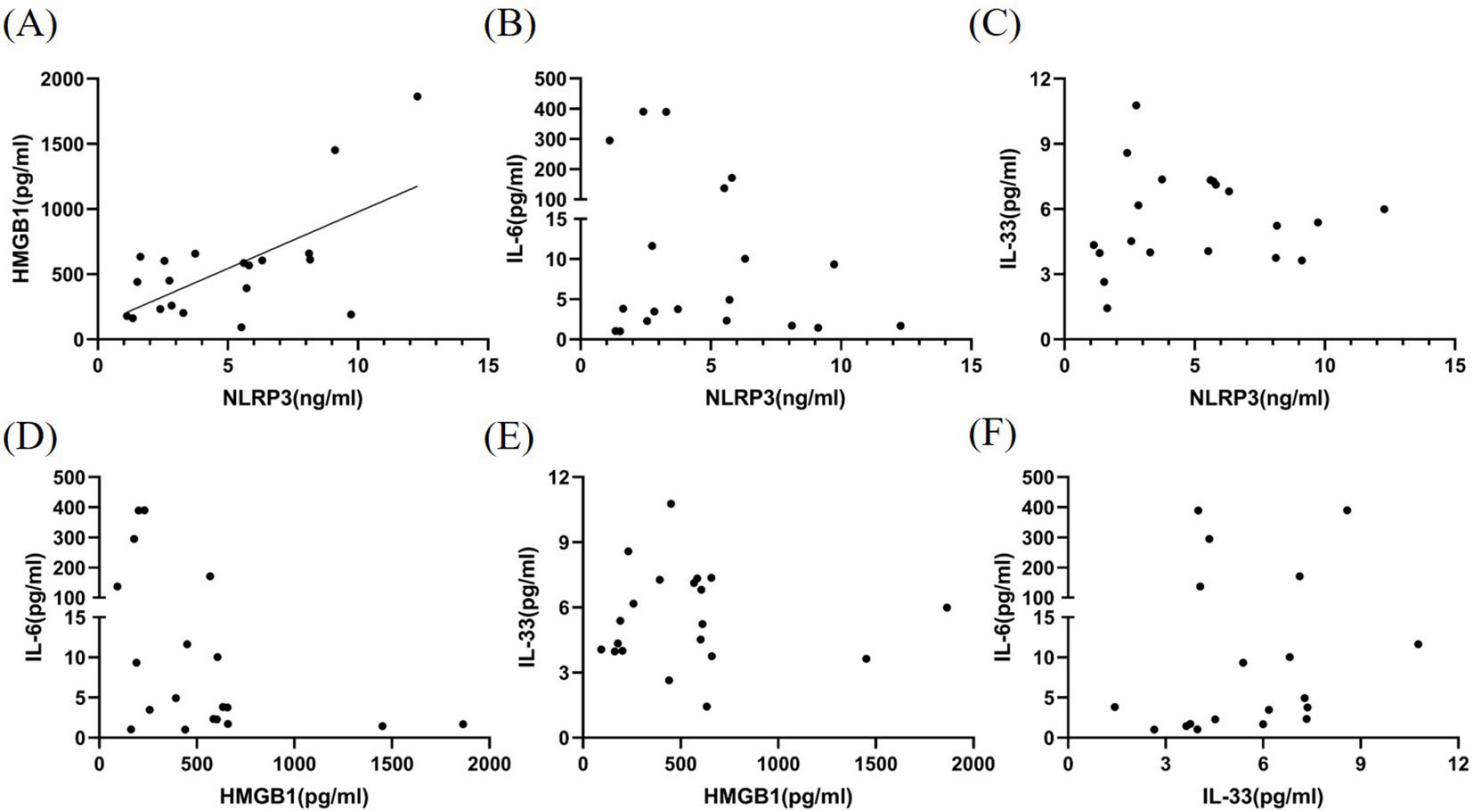
Correlation among NLRP3, HMGB1, IL-6, and IL-33 levels in the CSF of patients with MOGAD during the acute phase. HMGB1 levels **(A)** in the CSF were found to be significantly correlated with NLRP3 levels (*P* < 0.01, *r*² = 0.410). However, no significant correlations were observed among other inflammatory factors (*P*>0.05) **(B–F)**.

We examined the correlation among CSF NLRP3, HMGB1, IL-6, and IL-33 levels during the acute phase of MOGAD. HMGB1 levels in the CSF were found to be significantly correlated with NLRP3 levels (*P* < 0.01, *r* ^2^ = 0.410; **Figure 2A**). However, no significant correlations were observed among other inflammatory factors (*P*>0.05, **Figures 2B-F**).

### Relationship between disease severity and inflammatory factors in the CSF of patients with MOGAD during the acute phase

**Figure 3:**
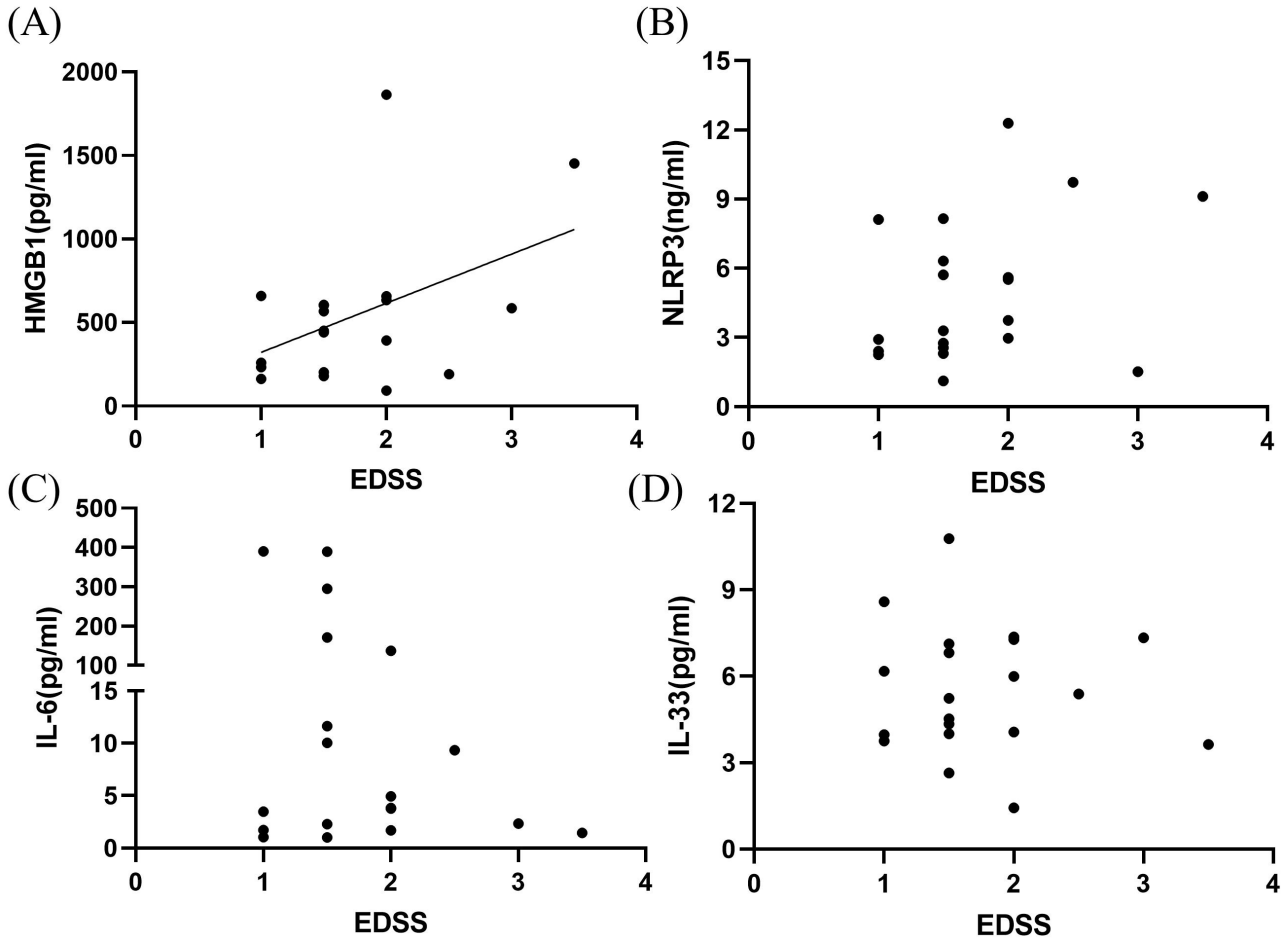
Correlation between inflammatory markers in the CSF and EDSS scores in patients with MOGAD during the acute phase. HMGB1 levels **(A)** were highly correlated with EDSS neural function scores (*P* < 0.05, *r*² = 0.200). However, no significant correlations were observed among CSF NLRP3 **(B)**, IL-6 **(C)**, IL-33 **(D)** levels and EDSS scores (*P* > 0.05).

EDSS scores were highly correlated with CSF HMGB1 levels (*P* < 0.05, *r* ^2^ = 0.200; **Figure 3A**). However, no significant correlation was observed among CSF NLRP3, IL-6, IL-33 levels and EDSS scores (*P* > 0.05; **Figures 3B–D**).

### Relationship between total number of attacks of the disease and CSF inflammatory factors in patients with MOGAD during the acute phase

**Figure 4:**
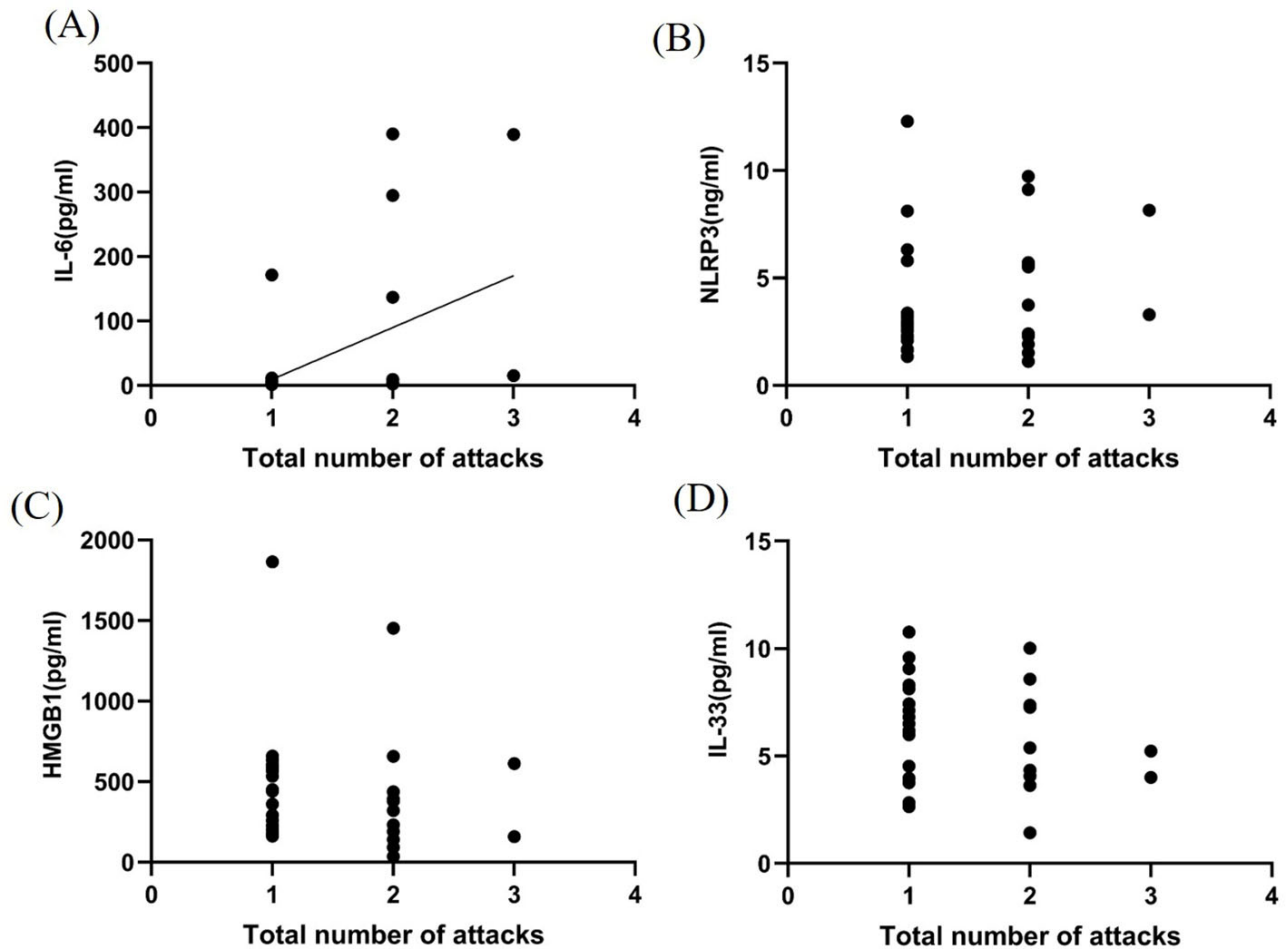
Correlation between inflammatory factors in the CSF and MOGAD recurrence during the acute phase. IL-6 levels **(A)** in the CSF were closely related to MOGAD recurrence (*P* < 0.05, *r*² = 0.204). However, no significant correlations were observed between NLRP3 **(B)**, HMGB1 **(C)**, and IL-33 **(D)** levels and number of attacks (*P* > 0.05).

The total number of attacks of the disease were positively correlated with CSF IL-6 levels (*P* < 0.05, *r* ^2^ = 0.204, **Figure 4A**). However, no significant correlations were observed between NLRP3 inflammasome, HMGB1, and IL-33 levels and number of attacks (*P* > 0.05, **Figures 4B–D**).

## Discussion/conclusion

Neuroinflammation plays a crucial role in the pathogenesis of demyelinating diseases of the CNS (10). Compared to serum, inflammatory factors in the CSF can more accurately reflect the inflammatory state of the CNS (11) and provide vital information. MOGAD is a unique syndrome of immunoinflammatory demyelinating diseases in the CNS (12). Neuroinflammation is accompanied by CSF and blood cytokines and chemokines, which play key roles in MOGAD (13). In this study, we found that children with MOGAD had higher levels of NLRP3, HGMB1, and IL-6 in the CSF during the acute phase than in the control group and remission. Additionally, HMGB1 was positively correlated with NLRP3 levels in the CSF of patients with MOGAD. Furthermore, HMGB1 levels in the CSF can reflect disease severity, and CSF IL-6 levels were found to be closely related to total number of attacks of the disease.

HMGB1, as a proinflammatory cytokine (14), can cause persistent neuroinflammation in the CNS (15). High HMGB1 levels can induce activated macrophages to release IL-6, IL-8 and other cytokines by activating NF-κB and enhance proinflammatory cytokine gene expression (16). Serum HMGB1 levels elevate in MS (6). However, changes in CSF HMGB1 levels in patients with MOGAD have not been studied. We found that HMGB1 levels in the CSF of children with MOGAD were significantly higher during the acute phase than those during the remission stage and in the control group, suggesting that the increase in HMGB1 levels may be an important factor that promotes the inflammatory response during the acute stage of MOGAD. Moreover, we found that HMGB1 levels in the CSF were strongly associated with disease severity. This may be due to the fact that HMGB1, as an immunostimulatory signal, induces dendritic cell maturation and proinflammatory cytokine secretion, leading to extensive demyelination and perivascular macrophage infiltration and neuronal swelling and necrosis. In addition to MOG-IgG titer, this finding also provides clinicians an opportunity to assess disease severity at an early stage, with individualized treatment strategies that can reduce the extent of neurological damage.

The nucleotide binding oligomerization domain-like receptor pyrin domain containing protein 3 (NLRP3) inflammasome is one of the most intensively studied inflammatosomes (17). The NLRP3 inflammasome is a complex composed of receptor protein NLRP3, connexin ASC, and effector protein caspase-1, which is involved in the secretion of many inflammatory factors. Once the NLRP3 inflammasome is activated, caspase-1 precursor protein secrets mature inflammatory cytokines IL-1 and IL-18 (18), induces immune response, and ultimately causes neuronal damage. The NLRP3 inflammasome is closely related to HMGB1 protein. Activation of the NLRP3 inflammasome has been reported to be associated with the release of HMGB1 protein in macrophages (19). HMGB1 activates the NLRP3 inflammasome through the TLR4/NF-κB signaling pathway, promoting the production of inflammatory cytokines such as IL-1β and TNF-α (18). HMGB1 might also affect the NLRP3 activation through the ROS/NF-κB pathway. This further makes the inflammatory response worse (20). Several studies have shown that serum NLRP3 expression is elevated in patients of MS, which is important for the early recognition of MS severity (21, 22). In experimental autoimmune encephalomyelitis (EAE) animal models, the inhibition of NLRP3 expression can also reduce the secretion of proinflammatory factors and alleviate spinal cord inflammation and demyelination damage (23). However, there has been limited research on changes in NLRP3 levels in the CSF of patients with MOGAD. We observed that NLRP3 levels in the CSF of children with MOGAD were significantly higher than those in remission and significantly correlated with HMGB1 levels. This suggests that NLRP3 and HMGB1 in the CSF are involved in the pathogenesis of MOGAD and important factors and promote the development of inflammation. There may be some molecular signaling pathways between NLRP3 and HMGB1 in patients with MOGAD that need to be further studied.

IL-6 plays an important role in the pathophysiology of MOGAD, and IL-6 blockers are a very promising treatment option for MOGAD (24). Tocilizumab, which is an IL-6 inhibitor, is a promising treatment for patients with recurrent MOGAD or those who do not respond to conventional immunotherapy (25). Elevated levels of IL-6 in the CSF and serum have been reported in patients with MOGAD (13, 26), which is consistent with our findings. In the present study, we observed that IL-6 levels in the CSF were strongly associated with MOGAD recurrence. This is possibly because IL-6 is involved in regulating humoral and cellular immune responses, including B cell differentiation, complement secretion, and T cell activation, thus leading to increased blood-brain barrier (BBB) permeability and triggering a sustained inflammatory cytokine storm (27).

HMGB1 activates the downstream NF-κB signaling pathway by binding to Toll-like receptor 4 (TLR4), which boosts the production of proinflammatory cytokines such as IL-6, thereby exacerbating the inflammatory response (28). In several inflammation models, activating NLRP3 increases IL-6 release, a process closely related to the activation of the NF-κB pathway (29). NLRP3 inhibition can notably reduce IL-6 levels, showing that NLRP3 is important for IL-6 production (30). Although both HMGB1 and NLRP3 can promote IL-6 production, no direct significant correlation was found between NLRP3 and IL-6, or HMGB1 and IL-6. This might be because of the inflammatory response occurring at different times and the small sample size, making it difficult to observe their direct link to IL-6. Moreover, different cell types can considerably change the interactions between HMGB1, NLRP3, and IL-6. Further, IL-6 might trigger set off negative feedback during inflammation, inhibiting its further release and production, and potentially complicating the observation of a direct link among them in some cases.

IL-33 is widely expressed in the human body and can produce inflammatory factors, affect the permeability function of the BBB (31), and cause functional impairment of the CNS (32). Significantly higher levels of IL-33 in the CSF and blood have been reported in patients with MS compared with healthy individuals (33). However, there are certain reports suggesting that IL-33 has neurorepair effects in the CNS (34). Administration of IL-33 can induce oligodendrocytes to develop and induce myelin cells, thereby reducing the severity of EAE (35). This suggests that IL-33 has a neuroprotective effect in MS (36). IL-33 can induce the proliferation of microglia and increase the expression of proinflammatory cytokines, such as TNF-α, IL-1β, and IL-10 (37). In this study, IL-33 levels in the CSF of patients with MOGAD during the acute phase were higher than those in the control group and lower than those in remission; however, no significant difference was observed between them, nor between CSF IL-33 levels and EDSS scores or recurrence, thus suggesting that IL-33 may play a dual role in the CNS. On the one hand, IL-33 may increase the expression of pro-inflammatory cytokines, affect the permeability of the blood-brain barrier, and cause tissue damage; On the other hand, excessive increase of IL-33 may induce oligodendrocyte development and myelin cells, playing a crucial role in nerve repair. The interaction between IL-33 and related inflammatory factors and the cytokine pathway needs to be further studied.

We found no direct significant correlation between IL-6 and IL-33. Even though IL-6 and IL-33 are involved in inflammation, their interaction can be affected by various biological processes. IL-6 primarily works through the JAK/STAT signaling pathway, boosting acute phase inflammatory responses (38). In contrast, IL-33 primarily activates Th2-type immune responses through IL-33R, boosting allergic responses and immune regulation (39). Furthermore, IL-6 has a broad impact on various immune cells (such as T cells, B cells, and macrophages), whereas IL-33 primarily affects Th2 cells and other specific cell types (40). This difference in target cells might explain why their interaction is not evident isn’t obvious, resulting in a lack of significant direct correlation. This difference in target cells might explain why their interaction is not evident, resulting in a lack of significant direct correlation.

In this study, we found that the levels of NLRP3, HMGB1, and IL-6 in cerebrospinal fluid (CSF) play an important role in myelin oligodendrocyte glycoprotein antibody-associated diseases (MOGAD). These findings enhance our understanding of MOGAD-related neuroinflammation in children. Furthermore, our results underscore the need for further exploration into the roles of HMGB1 and related signaling pathways as potential therapeutic targets in MOGAD. This research lays a foundation for future investigations into inflammatory cytokine pathways and highlights promising targets for the treatment of autoimmune diseases.

## Data Availability

The raw data supporting the conclusions of this article will be made available by the authors, without undue reservation.
